# Exchange rate asymmetry and its impact on bilateral trade: Evidence from BCIM-EC countries using N-ARDL approach

**DOI:** 10.1016/j.heliyon.2023.e23886

**Published:** 2023-12-19

**Authors:** Arifur Rahman, S. M. Woahid Murad, Xiaowen Wang

**Affiliations:** a School of Economics, Lanzhou University, Gansu Province, China; b School of Accounting, Economics and Finance, Curtin University, Perth, WA, Australia; c Department of Economics, Noakhali Science and Technology University, Noakhali, Bangladesh

**Keywords:** BCIM-EC, Bangladesh, N-ARDL, Asymmetric effect, Real exchange rate

## Abstract

The novelty of this study is to examine the asymmetric effect of the exchange rate on bilateral export and import between Bangladesh and its three trading partners in the Bangladesh-China-India-Myanmar Economic Corridor using nonlinear ARDL models from 1973 to 2022. After controlling income and structural breaks, the empirical findings confirm the asymmetric effects of exchange rates on the short-run and long-run export and import demand functions of Bangladesh. Furthermore, the impacts of the appreciation and depreciation of the Bangladeshi currency are heterogeneous for these three trading partners. For instance, the depreciation of the Bangladeshi currency increases exports to China and India while it decreases exports to Myanmar in the short run. However, the depreciation increases exports to India and Myanmar, and the appreciation also increases exports to China and India in the long-run. On the contrary, depreciation increases imports from China and Myanmar in the short-run, while it decreases imports from Myanmar in the long run. Only appreciation has significant negative effects on China and India. As a robustness measure, we exclude the COVID-19 period. However, it does not substantially change our main findings.

## 1 Introduction

The asymmetric effects of macroeconomic variables are indeed a phenomenon that have observed historically in the field of Economics. Like such prior empirical works [1] addressed an asymmetry within trade cycle. Building upon this foundation, the primary objective of this study is to assess the income and price elasticities within the export and import demand functions of Bangladesh, specifically concerning its trade relationships with China, India, and Myanmar. Since Bangladesh is a part and parcel of the BCIM-EC^1^ under “Belt and Road” initiative of China and India and China play crucial roles as major import trading partners of Bangladesh, they are of the utmost importance to this study.

Changes in exchange rates can certainly create asymmetric effects on trade flows. The asymmetric impact on trade flows from changes in exchange rates is influenced by several elements such as price elasticities of demand, the structure of a country's imports and exports as well as the international and domestic market competitiveness of industries. Additionally, other factors such as trade policies, market structures, and global economic conditions can further shape the extent of the asymmetric effects. See Refs. [2,3] for instance.

When the value of one nation's currency shifts relative to that of another nation's currency due to fluctuations in the exchange rate, it can have an uneven impact on the volume and direction of trade. The income elasticity of demand is a measure of how sensitive the demand for imports and exports is to changes in the overall level of income. Through the estimation of income elasticity, the study seeks to comprehend the effect of income changes, both in Bangladesh and the BCIM-EC countries, on bilateral trade flows. However, the study focuses on two main objectives. Firstly, it aims to examine the impact of Bangladesh's exchange rate on the bilateral import and export flows with its three trading partners in the BCIM-EC framework. Secondly, it seeks to estimate the income elasticity of demand functions for bilateral imports and exports between Bangladesh and the BCIM-EC member countries. By accomplishing these objectives, the study aims to provide valuable insights into the factors influencing trade dynamics and the effects of exchange rate fluctuations and income changes on trade flows within the BCIM-EC region.

Table 1 presents the combination of import and export sharing of Bangladesh with its three trading partners, namely China, India, and Myanmar and the world for the year 2022. Table 1 demonstrates that 40.60% of total import and 4.92% of total export occurred in the fiscal year 2022 within the BCIM-EC framework. The substantial volume of goods and services imported in Bangladesh from India and China is noteworthy, 40.43% of total import whereas total export with these two trading partners is 4.86%.

**Table 1:** Import and Export sharing of Bangladesh with its three trading partners (China, India, Myanmar) of BCIM-EC in 2022.

Country	Total Import (In million USD)	Import Sharing (%)	Total Export (In million USD)	Export sharing (%)
China	19599	24.45	570	1.22
India	12808	15.98	1707	3.64
Myanmar	141	0.17	29	0.06
Total	32548	40.60	2307	4.92
World	80162	100	46851	100

**Figure-1:**
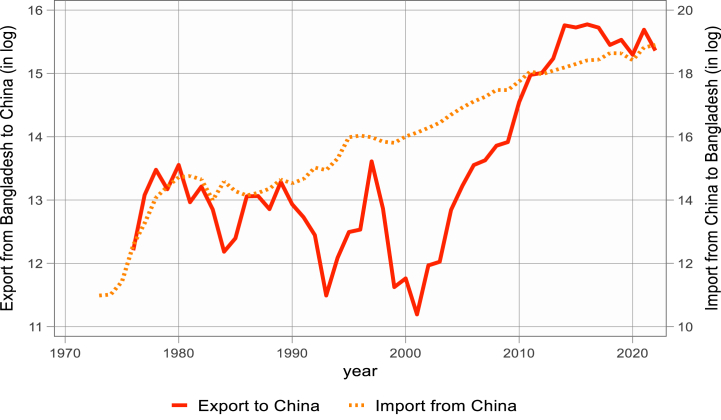
Bangladesh export to and import from China (1973–2022).

**Figure-2:**
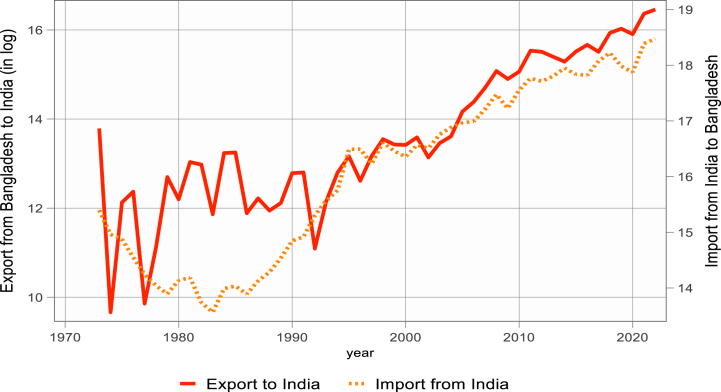
Bangladesh export to and import from India (1973–2022).

**Figure-3:**
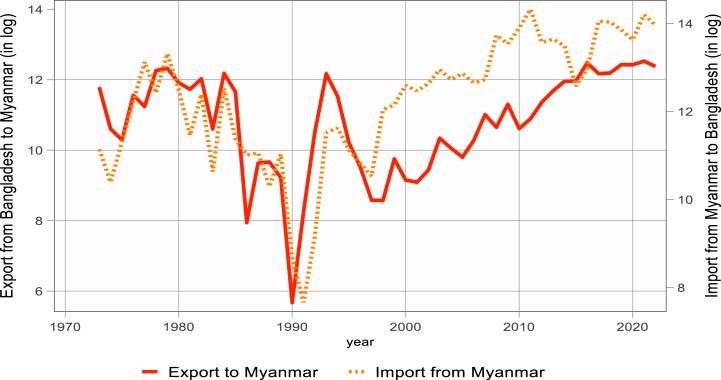
Bangladesh export to and import from Myanmar (1973–2022).

Source: Direction of Trade Statistics, IMF [4].

Figure-1, Figure-2, Figure-3 display the logarithmic representation of Bangladesh's export to and import from BCIM-EC countries over the last five decades. The volume of bilateral trade is increasing at an increasing rate. Over the past decade, Bangladesh has attained a notable average 6.5 % GDP growth. This remarkable performance has positioned the country among the top five fastest-growing economies globally. Additionally, in terms of purchasing power parity (PPP), Bangladesh ranks 31st, highlighting its economic strength. Furthermore, Bangladesh is recognized as one of the next-eleven emerging middle-income economies. However, the study investigates whether bilateral import and export demand functions of Bangladesh are significantly different with its three trading partners or not, by using Non-linear Auto-regressive Distributed Lag (N-ARDL) framework of [5].

Besides N-ARDL, this study disregards and considers structural breaks as a means of evaluating the changes in the underlying relationships over time. By adopting this methodology, the study aims to estimate the asymmetric effects of income and exchange rates on the demand for exports and imports, both in the short run and the long run. This consideration of asymmetry contributes to a more comprehensive understanding of the trade flow dynamics between Bangladesh and its trading partners within the BCIM-EC framework.

## 2 Review of literature

Traditional Heckscher-Ohlin theory of trade predicts trade equilibrium between two countries based on products specialties and natural resources [6,7]. According to the Linder hypothesis [8], countries with comparable income levels tend to engage in greater trade compared to those with disparate income levels and the nature of products consumption vary with the income level that ultimately leads to trade with each other. However, trade elasticities demonstrate the responsiveness of imports and exports to changes in economic activities (i.e., income) and relative prices (for instance: real exchange rate or RER). Trade elasticities were first focused on in the disciplines of Economics by Houthakker and Magee in 1969 [9]. Conventionally, trade elasticities are measured by analyzing comprehensive data, in which a country's total exports are calculated by regressing on against a foreign demand variable (such as trade-weighted foreign GDP) and relative price variables (such as the real effective exchange rate). Similarly, total imports are regressed against domestic GDP and the real effective exchange rate. Nevertheless, estimating trade elasticities in such a way is a challenging task due to aggregation bias and low variability of aggregate data or unavailability of real data. Bilateral trade data of trading partners are often used in place of aggregate data to eliminate these shortcomings.

[10] investigated the effects of the devaluation of currency for 14 developing and developed countries. The outcomes implied that a real devaluation of the UK pound would aggravate its Balance-of-Payment (BOP) and long-run trade balance [11]. study emphasizes that income effects tend to outweigh substitution effects, leading to a positive correlation between exchange rate volatility and bilateral trade [12]. has revealed that import and export demands of Bangladesh are inelastic regarding the exchange rate, whereas income elasticity is shown more than unity [13]. confided in Ref. [14] cointegration method and elicited that real depreciation of exchange rate is ineffective for the improvement of trade balance over the long-run [15]. have inquired into bilateral trade flows across the world and examined the effect of currency blocs together with exchange rate stability on trade.

[16] by conducting Johansen-Juselius cointegration technique for Bangladesh, uncovered that income elasticity was more significant than price elasticity [17]. have developed a comprehensive model to analysis the potentials of bilateral trade between Bangladesh and India [18]. conducted a study and investigated that the income elasticities were significantly larger than the price elasticities of fifteen major trading partners of Bangladesh. Hence, variations in income levels among Bangladesh's top fifteen trading partners have a significant positive impact on the country's exports [19]. has assessed Bangladesh's trade potentialities by analyzing panel data together with economic factor analysis like exchange rate, openness etc.

In the study conducted by Ref. [20] found that the import demand function of Bangladesh exhibits price inelasticity, while the demand function related to income elasticity demonstrates income elasticity [21]. estimated Bangladesh's bilateral export demand elasticities with its main export trading countries and the results described that for Germany, France and the United States export income elasticity of Bangladesh is positive and elastic. On the other hand, it is positive but inelastic, for the Belgium and the UK.

According to Ref. [22] their analysis on the purchasing power parity (PPP) between Bangladesh with China and India revealed that the exchange rate between these countries is not significantly influenced by the prices of foreign nations (China and India). However, they found that the domestic prices in Bangladesh exhibit a contrary pattern to what would be expected under the theory of PPP [23]. using annual data pertaining to Bangladesh from 1972 to 2006, examined the effects of currency depreciation on trade elasticities of Bangladesh and have examined positive effects of currency depreciation in the long-run and on the contrary mixed effects in the short-run [24]. using bilateral trade data examined Marshall-Lerner's (M-L) condition on a bilateral basis regarding six major trading countries (UK, U.S.A., India, Japan, Germany, and Hong Kong) of Bangladesh and found little evidence to support the M-L condition of Bangladesh [25]. using annual data of Bangladesh over the period of 1976–2009 and has found mixed outcomes in short-run and positive consequences of currency depreciation in the long-run [26]. conducted an empirical study that provided evidence that Bangladesh's exports to India are highly sensitive to changes in the country's competitiveness as measured by real exchange rate fluctuations [27]. investigated that the elasticity of income declines while the elasticity of the exchange rate rises in Bangladesh.

[28] investigated the correlation between Bangladesh's trade balance and the exchange rate using non-linear models. Incorporating a linear model provides support for the J-curve phenomenon. In the majority of cases, non-linear models have proven the existence of short-run asymmetry correction and the short-run asymmetry impacts due to variations in exchange rates [2]. employed the N-ARDL method to examine the trade balance between Malaysia and China across a range of 59 industries and have found that a substantial portion, approximately one-third, of these industries experienced significant effects due to Malaysian currency (Ringgit) depreciation against Chinese currency (Yuan). Another research of [29] has examined the asymmetric analysis and they found that non-linear ARDL models, most of the cases, supported the short-run asymmetric effects due to Bangladeshi currency (Taka) depreciation on trade balance with her eleven trading nations. Moreover, empirical studies also demonstrated that variations in the real exchange rate (RER) would have exaggerated bilateral trade balances for some trading countries but not for all trading countries, which indicates that the trend of the outcome of real exchange rate variations on the bilateral trade balance is still ambiguous [30]. described trade and investment potentiality among BCIM-EC by using Revealed Comparative Advantages (RCA) of Bangladesh, China, India, Myanmar trading block [31]. explored the trade and investment with geopolitical potentials of BCIM-EC member countries. They addressed the initiative of “One Belt One Road” (OBOR) as a next window for the development of the Asian economy.

Empirical studies on bilateral trade between Bangladesh and BCIM-EC member countries are very few [30,32,33] and most of the analyses are based on theoretical frameworks. Nevertheless, based on the literature review, none of the prior studies have investigated the asymmetric effect of exchange rate on bilateral trade of Bangladesh with these BCIM-EC member countries. Apparently, a research gap still exists in the empirical literature concerning the asymmetric effect of the exchange rate on bilateral trade, particularly when using the N-ARDL bounds test approach. We aim to address this gap by expanding on this through the inclusion of the N-ARDL cointegration approach of [5].

Finally, this paper aims to address a research gap by utilizing an econometric analysis, specifically the non-linear ARDL bounds testing approach to investigate the relationship between Bangladesh and its three major trading partners of BCIM-EC. The study aims to reveal the bilateral trade dynamics while ensuring the fulfillment of all essential advanced time-series properties.

## 3 Specification of models and methods

### 3.1 Unit root test

[34] proposed a Generalized Least Squares (GLS) based nonlinear unit root tests of a globally stationary smooth transition autoregressive (STAR) hereafter, exponential STAR (ESTAR) process, denoted to $\tilde{\omega }$, are specified by the Wald tests for $\varphi_{1}=\varphi_{2}=0$ in the subsequent regression:

(1) $$\Delta {\tilde{y}}_{t}^{x}=\varphi_{1}{\tilde{y}}_{t-1}^{x}1_{\left\{{\tilde{y}}_{t-1}^{x}\leq r_{1}\right\}}+\varphi_{2}{\tilde{y}}_{t-1}^{x}1_{\left\{{\tilde{y}}_{t-1}^{x}>r_{2}\right\}}+\sum_{i=1}^{k}\;\beta_{i}\Delta {\tilde{y}}_{t-i}^{x}+\varepsilon_{t}$$

In the following equation (1) where, x=μ for demeaned series and, x=τ for detrended series. $r_{1}$ and $r_{2}$ are the threshold factors, the lags of $\Delta {\tilde{y}}_{t}^{x}$ are measured to address the issue of serially correlated residuals.

Nonlinear unit root tests of this nature tend to exhibit more result accuracy compared to conventional ADF [35] unit root tests particularly when the data adheres to globally stationary SETAR or STAR processes^2^ [36]. The utilization of this method allows for the identification of nonstationary in relation to nonlinear processes within the variables being examined [34].

### 3.2 Specification of N-ARDL method

When estimating Bangladesh's export demand function [24], found that incorporating trading partners income and exchange rates were crucial components. Both factors, in particular, had substantial and statistically significant impacts on Bangladesh's export dynamics [9]. postulated that income of trading partners is critical element influencing export volume among them while determining the export demand function without taking the exchange rate into account and incorporating the income of trading partners as a key factor. On the other hand [37], considered the real exchange rate (RER) to estimate the bilateral import demand function. The findings of [38] pointed to income as the most significant element in Bangladesh's import and export demand. Import and export value indices based on bilateral trade are required to estimate the elasticities of bilateral import and export demand. However, owing to unavailability of such indices, the exchange rate is considered as an alternative. Consequently, the bilateral export and import demand functions of Bangladesh can be written as,

(2) $$\ln\;X_{r,t}=\beta_{0}+\beta_{1}\;\ln\;Y_{r,t}+\beta_{2}\;\ln\;{RER}_{r,t}+\varepsilon_{r,t}$$

(3) $$\ln\;M_{r,t}=\gamma_{0}+\gamma_{1}\;\ln\;Y_{t}^{BD}+\gamma_{2}\;\ln\;{RER}_{r,t}+\vartheta_{r,t}$$

Where, $X_{r}$ is Bangladesh's real export to the trading partner r and $M_{r}$ is Bangladesh's real import from the trading partner. Trading partner's real GDP is represented by $Y_{r,t}$, while the real GDP of Bangladesh is represented by $Y^{BD}$. ${RER}_{r}$ is the real exchange rate of Bangladeshi Taka against $r^{th}$ trading partner's currency. If this exchange rate increases (decreases), it implies that Bangladeshi Taka depreciates (appreciates) against the trading partner's currency. ε and ϑ are the white noise error terms. Finally, t is the time series operator. The income elasticities of export and import demand functions, $\beta_{1}$ and $\gamma_{1}$, are expected to be positive, while the exchange rate elasticity of export demand function ($\beta_{2}$) is expected to be positive and the exchange rate elasticity of import demand function ($\gamma_{2}$) is expected to be negative. However, contrary to the expectation, both $\beta_{1}$ and $\gamma_{1}$ might exhibit a reverse sign if an increase in income leads to rise in import substitute production, as suggested by Ref. [39].

The utilization of the N-ARDL model offers notable benefits and strengths in terms of effectively examining and assessing potential asymmetries that may exist within the relationships among variables. Therefore, it is feasible to utilize an empirical study employing an N-ARDL model to examine the potential variations in the way the dependent variable reacts to the independent variables in the presence of positive and negative shocks. However [40], found that fluctuations in exchange rates could have asymmetrical impacts on the trade balance between two countries. Likewise, we also consider the asymmetric effect of exchange rate in our model. To explore the asymmetric effect of exchange rate, positive and negative partial sums are generated in equation (4), where the positive and the negative partial sums stand for appreciation and depreciation of Bangladeshi taka in terms of the currency of the trading partner, respectively. Here, the threshold level is zero. Mathematically,

(4) $${POS}_{r,t}=\sum_{t=1}^{T}\Delta \ln\;{RER}_{r,t}^{+}=\sum_{t=1}^{T}\max\left(\Delta \ln\;{RER}_{r,t},0\right)$$

$${NEG}_{r,t}=\sum_{t=1}^{T}\Delta \ln\;{RER}_{r,t}^{-}=\sum_{t=1}^{T}\min\left(\Delta \ln\;{RER}_{r,t},0\right)$$

To obtain long-run and short-run coefficients within nonlinear ARDL framework of [5], substituting lnRER by the partial positive and negative sums of real exchange rate ${POS}_{r,t}$ and ${NEG}_{r,t}$ in Equations (2), (3) and, after taking some adjustments, the bilateral export and import demand functions are specified as follows:

(5) $$\Delta \ln\;X_{r,t}=\alpha_{0}+\sum_{j=1}^{a_{r}}\alpha_{1}\Delta \ln\;X_{r,t-j}+\sum_{j=0}^{b_{r}}\alpha_{2}\Delta \ln\;Y_{r,t-j}+\sum_{j=0}^{c_{r}}\alpha_{3}\Delta {POS}_{r,t-j}+\sum_{j=0}^{d_{r}}\alpha_{4}\Delta {NEG}_{r,t-j}+\rho_{1}\;\ln\;X_{r,t-1}+\rho_{2}\;\ln\;Y_{r,t-1}+\rho_{3}{POS}_{r,t-1}+\rho_{4}{NEG}_{r,t-1}+\omega_{r,t}$$

(6) $$\Delta \ln\;M_{r,t}=\lambda_{0}+\sum_{j=1}^{e_{r}}\lambda_{1}\Delta \ln\;M_{r,t-j}+\sum_{j=0}^{f_{r}}\lambda_{2}\Delta \ln\;Y_{r,t-j}^{BD}+\sum_{j=0}^{g_{r}}\lambda_{3}\Delta {POS}_{r,t-j}+\sum_{j=0}^{h_{r}}\lambda_{4}\Delta {NEG}_{r,t-j}+\gamma_{1}\;\ln\;M_{r,t-1}+\gamma_{2}\;\ln\;Y_{r,t-1}^{BD}+\gamma_{3}{POS}_{r,t-1}+\gamma_{4}{NEG}_{r,t-1}+u_{r,t}$$

Equations (5) and (6) can be explained according to Ref. [41] ARDL model with bounds testing approach. Here, Δ indicates 1st difference series; $\alpha_{0}$ and $\lambda_{0}$ are the drift terms; $a_{r}$ to $h_{r}$ are the lag lengths selected by the Akaike information criterion (AIC). The short-run estimators are $\alpha_{1}$ to $\alpha_{4}$ and $\lambda_{1}$ to $\lambda_{4}$, while the long-run estimators are $\rho_{1}$ to $\rho_{4}$ and $\gamma_{1}$ to $\gamma_{4}$. The long-run coefficients of export and import demand functions are normalized on $\rho_{1}$ and $\gamma_{1}$, respectively. The error terms are presented by $\omega_{r,t}$ and $u_{r,t}$. It is possible to have short-run estimates when the variables ΔPOS and ΔNEG take different lag order. If sign or size of coefficients gained from ΔPOS which is dissimilar from the scope or sign of the same factors found for ΔNEG at the similar lag, then short-run estimates effects are established.

However, if $\sum \alpha_{3}\neq \sum \alpha_{4}$, there would be an absolute sign for short-run asymmetric impacts of exchange rate on export. Finally, long-run asymmetric effects are subject to the following condition $\rho_{3}\neq \rho_{4}$. Similarly, if $\sum \lambda_{3}\neq \sum \lambda_{4}$, there would be an absolute sign for short-run asymmetric impacts of exchange rate on import and the long-run asymmetric effects hold if $\gamma_{3}\neq \gamma_{4}$.

## 4 Results and discussion

### 4.1 Unit root test

To identify the existence of integration of the variables particularly non-linear relationship, we have applied Kapetanios & Shin (2008) GLS detrending-based nonlinear unit root test as proposed by Ref. [34]. The results of the [34] nonlinear unit root test are presented in Table 2. We assume (as our “null hypothesis”) that, the variable under consideration has a unit root or is non-stationary, and we also assume (as our “alternative hypothesis”) that it does not. Variables contain a unit root in their levels, but all of the variables (income, real exchange rate, export, import) become stationary at *I* (1) after taking the first difference. Consequently, we can proceed to nonlinear ARDL model.

**Table 2:** Kapetanios & Shin (2008) GLS based nonlinear unit root test [34].

		Detrended series			
Country	Variable	Lags	KS-stat. (level)	Lags	KS-stat. (1st difference)
India	lnY	0	−1.927	1	−3.986***
	lnRER	3	−1.487	1	−7.866***
	lnX	3	−1.257	0	−3.262***
	lnM	3	−1.207	0	−3.944***
China	lnY	1	−1.800	0	−3.051*
	lnRER	3	−1.731	2	−3.876**
	lnX	0	−1.750	1	−3.597**
	lnM	3	−2.811	3	−4.823***
Myanmar	lnY	2	−1.555	0	−5.456***
	lnRER	0	−1.589	0	−5.732***
	lnX	0	−2.967	0	−7.095**
	lnM	0	−1.879	0	−4.217***
Bangladesh	lnY^BD^	3	−0.474	1	−3.761**

Notes: *, **, and *** indicate that the null hypothesis is rejected at significance levels of 10 %, 5 %, and 1 %, respectively. Lag-length are based on AIC criteria. Y denotes income, RER denotes real exchange rate, X represents export, M represents import.

### 4.2 Structural breaks

We applied the LM unit root test proposed by Ref. [42] to determine if our data exhibits a unit root problem when two endogenous structural breaks are present. Table 3 presents the results of [42] unit root test. According to this result, all of the variables are stationary with two endogenous structural breaks either in level or first difference. Therefore, both [34,42] unit root tests provide the basis of nonlinear ARDL model. To estimate nonlinear ARDL model allowing two breaks, we generate two dummy variables according to the findings of Table 3 and include them in Equations (5) and (6) accordingly. For instance, the structural breaks of bilateral export from Bangladesh to India are 1994 and 2005, while these breaks are 1993 and 2001 for import demand function. The dummy variable TB (time break) is equal to 1 when t ≥ (breakdown year plus one) if not TB (time break) = 0. To obtain robust outcome, we estimate N-ARDL bounds test by considering and disregarding structural breaks.

**Table 3:** Lee and Strazicich (2003) structural breaks test.

Country	Variable	t-statistics	TB1	TB2	Lags
India	lnY	−6.433**	1990	2011	5
	lnRER	−4.093	1984	1996	4
	ΔlnRER	−6.132*	1983	2003	1
	lnX	−6.956**	1994	2005	6
	lnM	−4.456**	1983	2001	5
China	lnY	−5.820*	1983	2012	5
	lnRER	−5.924*	1984	1995	1
	lnX	−7.755***	1998	2009	3
	lnM	−6.042*	1984	2008	3
Myanmar	lnY	−6.130**	1994	2010	6
	lnRER	−9.639***	1984	2010	6
	lnX	−6.602**	1987	1996	3
	lnM	−6.157**	1986	1995	3
Bangladesh	lnY^BD^	−4.899	1997	2010	4
	ΔlnY^BD^	−6.034**	1998	2012	5

Notes: *, **, and *** indicate that the null hypothesis is rejected at significance levels of 10 %, 5 %, and 1 %, respectively. First and second structural break is represented by TB1 and TB2, respectively. Δ is the 1st difference operator.

### 4.3 N-ARDL bounds test

The N-ARDL bounds test is performed by using [5] bounds test approach. We have used [41] critical values to identify whether the variables are cointegrated or not. In the bounds testing cointegration approach, the null hypothesis postulates that there is no cointegration between the variable under consideration. The alternative hypothesis suggests that the variables are cointegrated.

The *F*-statistics value is compared to the upper bound *I*(1) critical value to ascertain the presence of a cointegrating relationship, which is indicative of a long-term association between the variables. In contrast, if the *F*-statistics is lower than the lower bound *I*(0) critical value, we can infer that there is no cointegration between the variables under consideration, indicating the absence of a long-run relationship. Finally, the result will be inconclusive, if F-statistic is in between lower and upper bounds. However, in case of inconclusive results, the N-ARDL can still be used for determining the existence of a long-run relationship between variables, following [43,44].

### 4.4 Empirical results of N-ARDL bounds test

The N-ARDL procedures have been implemented to determine the parameters and examine the effect of the real exchange rate and real income on the export and import of Bangladesh with BCIM-EC member countries by testing the cointegration methods of the N-ARDL [5]. Table 4–5, report the results of the nonlinear ARDL, following Equations (5) and (6). The short-run and long-run estimation results are presented in Panel I and Panel II, respectively. Panel III is for diagnostic statistics. The analysis was implemented by using two-time breaks (TB_1_ and TB_2_). TBx and TBm denote time break for export and import, respectively. It is anticipated that significant historical events, such as the bearish stock market in 1987 that resulted in an economic downturn, the Asian financial crisis in 1997, the global financial crisis in 2008, and the COVID-19 pandemic in 2020 etc. may have a substantial influence on the trajectory of relative incomes, imports, and exports over time. In light of this, the incorporation of two structural breaks have the potential to enhance the accuracy and precision of our findings. The results indicate that the cointegration relationship is lower when there are no structural breaks and higher after imposing structural breaks.

**Table 4:** N-ARDL results of **Export** with and without structural breaks of China, India and Myanmar (1973–2022).

		China							India						Myanmar		
**Panel I: Short-run Export Coefficients** (Without Break) rowhead																	
Variable\Lag order		0	1		2		3		0		1		2		0	1	
Δx			.41** (.19)		.46** (.19)		.49** (.18)										
Δy		−1.25 (3.17)	−7.69** (3.63)		7.29** (3.13)				2.70*** (.78)						−5.16* (2.62)	−2.95 (2.97)	
ΔPOS		2.60* (1.44)	.89 (1.42)		2.62** (1.26)		1.54 (1.12)		−2.21 (2.23)		.394 (.69)		−2.65*** (−3.36)		−3.44*** (.65)		
ΔNEG		.914 (1.05)	−4.11*** (1.06)		−1.73 (1.04)		−3.40*** (.90)		1.14** (.49)						.026 (.143)		
(With Break) rowhead																	
Δx			.29* (.17)		.66*** (.14)		.52*** (.12)								.19 (.12)		
Δy		−1.76 (2.51)	−5.87** (2.83)		9.75*** (2.52)				−4.55 (2.91)						1.10** (.42)		
ΔPOS		3.09** (1.21)	1.57 (1.20)		.40 (1.07)		2.45** (.91)		1.40*** (.45)						−1.72*** (.51)		
ΔNEG		1.51 (.93)	−2.10** (.73)						1.54** (.68)						−.15 (.09)		
ΔTBx1		−.54 (.41)	−1.11** (.48)						1.19** (.47)						1.16 (.97)	−.84 (.78)	
ΔTBx2		−.39 (.38)	−.03 (.39)		−.02 (.39)		−.88** (.38)		.77* (.41)						−1.74*** (.48)		
**Panel II: Long-run Export Coefficients** (Without break)										**Panel III: Diagnostic Tests**							
Country\Variable	Constant		y	POS		NEG	TBx1	TBx2		${WT}_{SR}$		${WT}_{LR}$		F	${ECT}_{t-1}$	$R^{2}$	Adj. $R^{2}$
China	−39.38* (22.01)		1.84** (.83)	.53 (.97)		3.27*** (.63)	–	–		13.0***		3.03*		7.23***	−1.01*** (.21)	0.65	0.40
India	−60.37*** (18.47)		4.12*** (1.18)	−2.46 (1.83)		1.75** (.74)	–	–		2.60		3.19*		6.42***	−.65*** (.14)	0.56	0.48
Myanmar	−34.16 (13.44)		2.48*** (.72)	−3.89*** (.58)		.030 (.163)	–	–		24.63***		35.09***		13.69***		0.68	0.58
(With break) rowhead																	
China	−26.29 (24.81)		1.24 (.75)	.54 (.79)		1.58** (.65)	−1.10 (.32)	.839** (.384)		9.14***		0.56		6.48***	−1.15*** (.25)	0.87	0.69
India	−25.26 (18.31)		1.32** (.64)	1.36** (.51)		1.49** (.63)	1.15** (.46)	.752* (.406)		0.02		0.04		17.78***	−1.03*** (.12)	0.72	0.68
Myanmar	−14.32 (9.07)		1.44 (.52)	2.24*** (.78)		−.203 (.12)	.90 (.98)	−2.279*** (.685)		8.91***		6.64**		9.02***	−.76*** (.13)	0.71	0.63

Notes: The figures in parenthesis represent standard errors. *, **, and *** indicate significance level of 10 %, 5 %, and 1 %, respectively.

**Table 5:** N-ARDL results of **Import** with and without structural breaks for China, India and Myanmar (1973–2022).

	China				India				Myanmar			
**Panel I: Short-run Export Coefficients (Without Break)**												
Variable\ Lag order	0	1	2	3	0	1	2	3	0	1	2	3
Δm		−.0006 (.37)	.54** (.24)	.25 (.22)		−.08 (.12)	−.36** (.13)			.29** (.14)		
$\Delta y_{BD}$	4.03 (3.54)	−3.08 (3.32)	−2.86 (3.16)	9.60*** (2.63)	1.89 (2.42)	−4.28* (2.50)	.15 (2.09)	3.128 (1.74)	−12.45 (10.01)	8.70 (10.87)	3.23 (10.83)	40.29*** (11.34)
ΔPOS	1.64** (.73)	−1.33** (.61)	−.74 (.53)	−1.02* (.51)	.63 (.43)				−.17 (1.94)	3.42* (1.76)		
ΔNEG	−.061 (.40)	−1.53*** (.32)	.003 (.33)	.20 (.38)	−.93*** (.19)				.133 (.16)	−.27 (.17)		
(With Break)												
Δm		.27* (.15)	.31** (.12)	.25** (.11)		−.10 (.14)	−.40** (.16)			.96*** (.23)	.57** (.19)	.30 (18)
$\Delta y_{BD}$	.82** (.42)				1.78 (2.41)	−2.68 (1.69)			−2.21 (9.91)	.325 (10.67)	−10.25 (10.92)	25.56* (12.98)
ΔPOS	.25 (.52)				.54 (.45)				.463 (2.89)	5.82** (2.38)		
ΔNEG	.90 (.38)	−.73** (.35)			−.98*** (.17)				.08 (.13)	−.44*** (.14)	−.26* (.14)	
ΔTBm1	.73*** (.24)								−.72 (1.25)	1.43 (1.24)	2.33** (.95)	2.90*** (.75)
ΔTBm2	.17 (.13)								.09 (.79)	−1.41** (.68)	−.99* (.56)	
**Panel II: Long-run Import Coefficients** (Without break)_							**Panel III: Diagnostic Tests**					
Country \Variable	Constant	$y_{BD}$	POS	NEG	TBm1	TBm2	${WT}_{SR}$	${WT}_{LR}$	F	${ECT}_{t-1}$	$R^{2}$	Adj. $R^{2}$
China	47.59 (18.75)	−1.88** (.95)	3.07 (.81)	−1.11 (.32)	–	–	3.54*	15.12***	8.06	−.91*** (.40)	0.91	0.76
India	10.40 (7.19)	−.71 (1.17)	1.70 (1.57)	−2.49*** (.55)	–	–	12.39***	4.26**	8.33**	−.37*** (.12)	0.62	0.51
Myanmar	−70.03** (27.22)	3.84*** (1.04)	−2.22*** (.74)	.38*** (.12)	–	–	2.21	11.48***	9.10***	−.86*** (.16)	0.65	0.50
(With break)												
China	−13.02 (10.38)	1.48 (1.02)	.46 (.84)	−.68*** (.23)	−.10 (.26)	.31 (.23)	1.02	1.49	6.11*	−.55*** (.15)	0.70	0.59
India	7.50 (7.40)	1.43 (1.60)	1.43 (1.60)	−2.59*** (.72)	−.33 (.42)		12.38***	3.21*	10.67*	−.38*** (.13)	.60	.50
Myanmar	−114.59*** (38.34)	3.08*** (.62)	−1.63** (.88)	.23*** (.06)	−1.33** (.49)	1.23*** (.24)	2.51	4.64**	11.19***	−1.85*** (.31)	0.88	.71

Notes: The figures in parenthesis represent standard errors. *, **, and *** indicate significance level of 10 %, 5 %, and 1 %, respectively.

We have found different N-RADL estimation results for different coefficients. In the short-run estimates with structural breaks, the export coefficient of real income is significant for China and Myanmar. It means that in the short-run, an increase in the real income of China and Myanmar will increase export to those countries but not to India. Based on the N-ARDL without break, the results are statistically significant for China and India. However, short-run results do not remain valid for the long-run when considering structural breaks except for India. For example, in the long-run estimation through the N-ARDL with break, there is a positive and significant result at the 5 % significance level between real income and export to India. Therefore, in the case of the N-ARDL with break, our long-run estimation result for India is significant and has a positive relationship between real income and export to India. However, the long-run N-ARDL without break results are significant at 5 % significance level for china and 1 % significance level for India and Myanmar. Therefore, the study results show a mixed effect in the case of real income.

Our expected income elasticity for export $\left(\beta_{1}\right)$ is positive. For long-run estimation results with structural breaks, we found a positive expected relationship for India but not for China and Myanmar. According to empirical results, the relationship may vary depending on each country's economic and social characteristics. For example, proper infrastructural development [45,46], economic development, and abundance of natural resources [47] etc.

Our expected sign of the exchange rate elasticity of the export demand function $\left(\beta_{2}\right)$ is positive. Based on N-ARDL, disregarding breaks for export, the real exchange rate (RER) is significant and has a positive (ΔPOS) effect (exchange rate appreciation) on China, India, and Myanmar in the short-run, but it is significant but negative only for Myanmar in the long-run. On the other hand, based on N-ARDL, considering breaks, the results are significant in the short-run with the exception of China in the long-run. However, the long-run positive effect of the exchange rate on Bangladesh's export is negative but robust only for Myanmar in disregarding breaks, positive and significant for India and Myanmar after considering structural breaks.

For short-run estimation with and without structural breaks, we found the coefficients of the *ΔNEG* variable is positive and significant in the case of India only. It means that in the short run, the depreciation of the Bangladeshi currency will increase export to India but not to China and Myanmar. However, it is negative but significant for China if it is not considered structural breaks. On the other hand, when considering structural breaks, we find a negative but statistically significant result for China.

According to N-ARDL with break, the long-run results of the export coefficient of *NEG* variable show significant results for India and China but not for Myanmar. It implies that in the long run, the depreciation of the Bangladeshi currency will increase export to India and China but not to Myanmar. Therefore, the mixed results of *ΔPOS* and *ΔNEG'*s coefficients, show asymmetry. The results are also supported by the short-run and long-run Wald tests (denoted by *WT*_*SR*_ and *WT*_*LR*_)*,* except for India, while not considering structural breaks. However, results are also supported by F test as it is statistically significant at 1 % significance level in both cases. The income elasticity of import demand functions ($\gamma_{2}$) is expected to be positive, while the exchange rate elasticity of import demand functions is expected to be negative. In the short-run estimation with and without structural breaks, the import coefficient of real income (Bangladesh) is significant and positive for China and Myanmar. For India, it is significant but negative in the absence of structural breaks. However, in the long-run, it is significant and positive only for Myanmar and significant but negative for China.

According to N-ARDL, with breaks for import, the real exchange rate (RER) is significant (*ΔPOS* is significant and positive) for China (at the immediate effect) and Myanmar in the short run. It implies that an appreciation of the Bangladeshi currency will increase import from China and Myanmar in the short-run. However, it is significant but negative for Myanmar and lacks significance for India and China in the long-run. The coefficients of the *ΔNEG* (depreciation of Bangladeshi currency) variable are significant for India and China at any short-run consideration but significant for Myanmar after considering structural breaks. However, the short-run result will remain valid in the long-run, except for China, in case of disregarding structural breaks. The short-run coefficients of *ΔPOS* and *ΔNEG* display asymmetry, as evidenced by the inconsistent findings. The results are further validated by the short-run Wald test (*WT*_*SR*_) with the exception of Myanmar, when structural breaks are not considered. However, the findings are further substantiated by the long-run Wald test (denoted by *WT*_*LR*_), with the exception of China, when accounting for structural breaks. The F test results also support the findings in both the short-run and long-run, except for China, when there are no structural breaks. The short-run coefficients of import and export (*m* and *x*) are all statistically significant at different levels. However, in the context of export and import, our research findings don't find unidirectional exchange rate effect on bilateral trade, as the results show mixed effect while considering and disregarding structural breaks in the short-run and the long-run.

Likewise Table 4–5, Table 6–7 report the results of the nonlinear ARDL without structural breaks. The short-run and long-run estimation results are demonstrated in Panel I and Panel II, respectively. Panel III is for diagnostic statistics. More specifically, Table 6 and 7 provide insights into the pre-COVID situation (1973–2019), with a particular focus on the absence of structural breaks. In the context of export analysis, it is noteworthy to observe that differing coefficients with distinct levels of significance emerge when considering and disregarding the COVID-19 period, with the exception of India. The export coefficient of real income is found to be significant when considering the COVID-19 period (Table 4) in short-run estimates, but it is not significant when disregarding the COVID-19 period (Table 6). However, in the short-run, we get a positive and significant result of *ΔPOS* coefficient for India if we disregard the COVID-19 period (Table 6), but the short-run and the long-run Wald tests don't support it.

**Table 6:** N-ARDL results of Export without structural breaks for China, India, and Myanmar (1973–2019).

Panel I: Short-run Export Coefficients																	
	China						India	Myanmar									
Variable\Lag order	0		1	2		3	0	0		1	2		3	4		5	
Δx			.51*** (.18)	.55*** (.18)		.43** (.17)											
Δy	−8.62** (3.24)						−5.39 (3.92)	−8.74* (4.97)		−5.23 (6.59)	14.84** (6.27)		5.69 (6.66)	−2.83 (7.58)		−27.33*** (7.27)	
ΔPOS	3.50** (1.44)		1.06 (1.43)	2.90** (1.24)			1.03** (.48)	−3.63*** (.68)									
ΔNEG	.318 (1.03)		−4.54*** (1.01)	−1.90* (.98)		−3.48*** (.87)	.91* (.48)	.06 (.15)									
**Panel II: Long-run Export Coefficients**									**Panel III: Diagnostic Tests**								
Country\Variable		Constant		y	POS		NEG	$W_{SR}$		$W_{LR}$	F	${ECT}_{t-1}$			$R^{2}$		Adj. $R^{2}$
China		−27.15 (20.84)		1.29* (.75)	1.22 (.90)		2.95***	19.72***		1.47	8.97***	−1.10*** (.19)			.65		.43
India		−43.73*** (15.41)		2.08*** (.49)	1.03* (.55)		.92** (.45)	0.02		0.02	20.05***	−1.00*** (.14)			.67		.63
Myanmar		−42.07*** (14.47)		2.85*** (.75)	−4.01*** (.58)		.07 (.16)	26.20***		38.19***	14.01***	−.91*** (.13)			.71		.61

Notes: The figures in parenthesis represent standard errors. *, **, and *** indicate significance level of 10 %, 5 %, and 1 %, respectively.

**Table 7:** N-ARDL results of Import without structural breaks for China, India, and Myanmar (1973–2019).

**Panel I: Short-run Import Coefficients**																						
	China											India					Myanmar					
Variable\Lag order	0	1		2		3	4	5		6		0	1	2	3		0		1	2		3
Δm		−.56		.36		.04	.46	.66**		.18			.027	−.26*	.22				.34**	.19		
		(.48)		(.34)		(.33)	(.26)	(.25)		(.21)			(.16)	(.15)	(.15)				(.15)	(2.00)		
$\Delta y_{BD}$	2.62	−9.59**		−7.97**		6.44**	7.01	−9.56**				.34					−22.57*		3.40	−3.87		41.29***
	(4.86)	(4.12)		(3.01)		(2.80)	(4.24)	(3.73)				(.52)					(11.79)		(13.31)	(13.10)		(11.55)
ΔPOS	1.95**	−.87		−1.07		−1.33**	−2.64**					.01					3.58*					
	(.88)	(1.07)		(.82)		(.53)	(.86)					(.56)					(1.89)					
ΔNEG	.62	−1.67***		−.35		.47	.43	1.68***		.54		−.83***					.16		−.26			
	(.48)	(.39)		(.38)		(.41)	(.36)	(.45)		(.34)		(.23)					(.16)		(.17)			
**Panel II: Long-run Import Coefficients**									**Panel III: Diagnostic Tests**													
Country\variable	Constant		$y_{BD}$		POS		NEG		$W_{SR}$		$W_{LR}$		F			${ECT}_{t-1}$		$R^{2}$			Adj. $R^{2}$	
China	25.94		−1.03		2.75**		−1.21**		5.09**		7.03**		5.57**			−.78***		.92			.73	
	(31.05)		(1.47)		(1.23)		(.46)									(.49)						
India	−2.07		.75		.03		−1.84***		1.96		1.28		4.70**			−.45***		.54			.45	
	(10.44)		(1.00)		(1.25)		(.48)									(.14)						
Myanmar	−87.22**		4.47***		−2.44***		.38***		0.02		13.80***		8.90***			−.92***		.69			.55	
	(31.66)		(1.09)		(.73)		(.12)									(.17)						

Note: Notes: The figures in parenthesis represent standard errors. *, **, and *** indicate significance level of 10 %, 5 %, and 1 %, respectively.

The import scenario is quite different between the pre-COVID-19 and considering COVID-19 period. For the coefficient of real income, we get a negative but significant result for China and Myanmar, with the exception of India. For long-run real exchange rate coefficients, POS and NEG have significant results only for China (Table 7) as compared with considering the COVID-19 period (Table 5), where they show lack of significance. The long-run Wald test and F test also support the results.

The speed of adjustment or error correction term measures how quickly the dependent variable converges to long-run equilibrium [44]. Obtaining significantly negative coefficients for ${ECT}_{t-1}$ would provide support for the presence of cointegration. ${ECT}_{t-1}$ is negative and significant at the 1 % significance level both in considering and disregarding the Covid-19 period, indicating that the coefficient would get back to the long-run equilibrium. The adjusted $R^{2}$ measures the quality of the fit of the regression model. The diagnostic statistics value, specially adjusted $R^{2}$, is greater when the structural breaks are incorporated, indicating that it increases the goodness of fit of our models.

### 4.5 Recursive CUSUM tests without and with break

**Figure-4:**
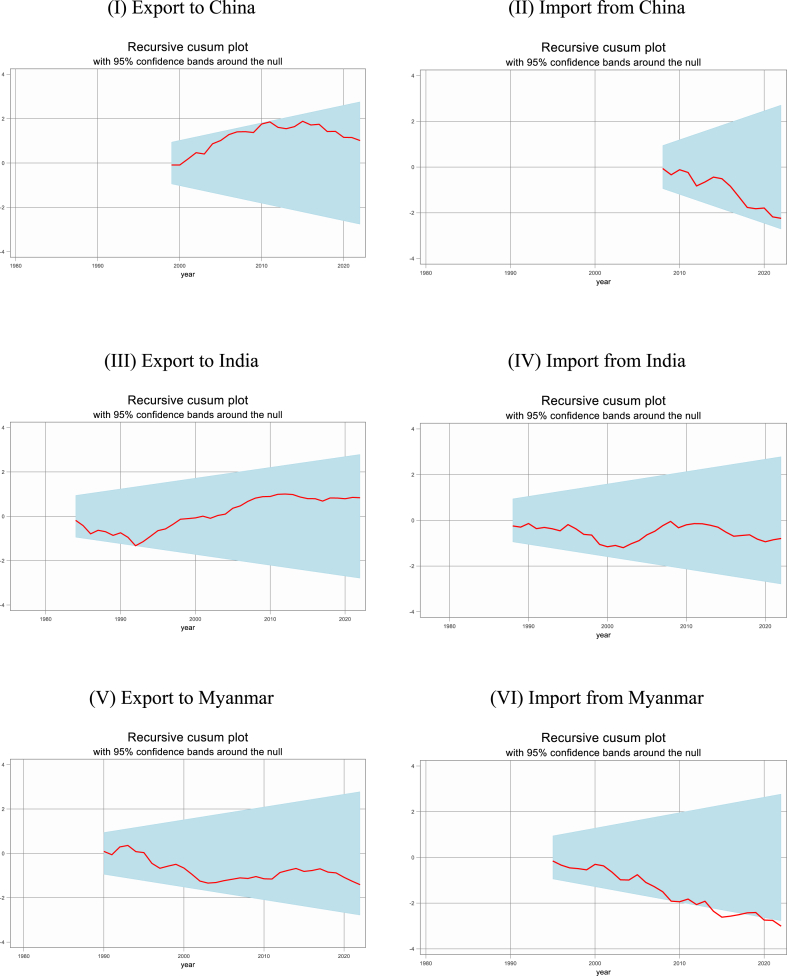
Recursive CUSUM test without break.

**Figure-5:**
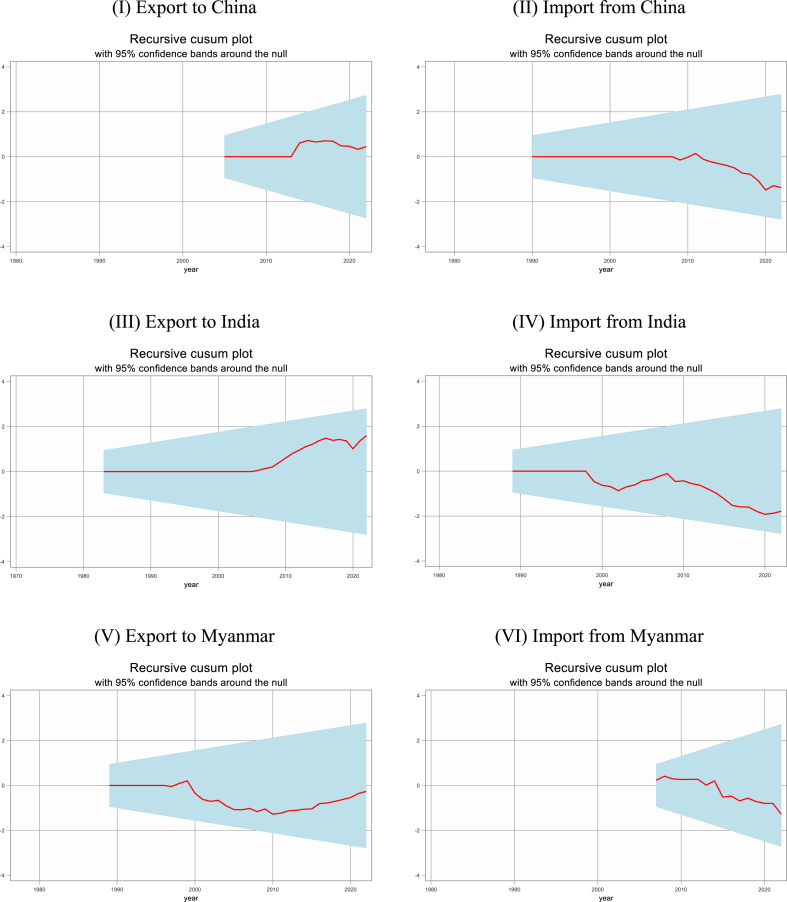
Recursive CUSUM test with break.

The study further conducted recursive cumulative sum (CUSUM) tests without and with break as shown in Figure-4, Figure-5. The results demonstrate that structural stability exists for both short-run and long-run elasticities for the residuals of both export and import demand functions. The recursive CUSUM plot with 95 % confidence bands around the null for the nonlinear model demonstrates that, the estimated short-run and long-run coefficients are stable over time.

## 5 Conclusion

The involvement of Bangladesh in the Bangladesh-China-India-Myanmar Economic Corridor (BCIM-EC) initiative, along with the significant contributions of India and China as key import trading partners for Bangladesh underscore them to consider into our empirical study. The study considers annual disaggregated data from 1973 to 2022 while considering and disregarding structural breaks. Based on N-ARDL bounds testing approach, we found different cointegration results before and after structural breaks. The study finds a significant positive correlation between real income and short-run export to China, India, and Myanmar. It is also observed that the effects of this relationship exhibit variations over the long-run. Regarding the exchange rate, it is important to acknowledge that the depreciation of Bangladeshi currency can impact export differently among BCIM-EC countries. In the short-run, currency depreciation has been found to have a positive effect on export to India compared to China and Myanmar. In the long-run, there are noticeable tendencies towards improving export to India and China, but this trend is not observed in the case of Myanmar. On the other hand, real income significantly affects the import of China and Myanmar, particularly in the short-run. However, Myanmar consistently maintains the correlation between real income and imports over the long-run.

Our empirical research has checked the robustness by considering COVID-19 (1973–2022) and disregarding it (1973–2019), focusing the results on the absence of structural breaks. Export analysis shows different coefficients and distinct levels of significance, with India being the exception. Import scenarios differ between pre-COVID-19 and considering COVID-19 periods, with negative but significant results for China and Myanmar. The long-run real exchange rate coefficients POS and NEG have significant results for China, which differ from the results of considering the COVID-19 period. The long-run Wald tests and the *F* tests also support the results. The speed of adjustment term, ECT_t-1_, shows significant negative coefficients, indicating long-run adjustments.

The implementation of well-balanced monetary and fiscal policy have the potential to significantly enhance the facilitation of bilateral trade in Bangladesh. Regarding export, monetary policy plays a crucial role in regulating the money supply and managing exchange rates, whereas fiscal policy primarily aims to stimulate economic growth by investing in infrastructure and increasing government spending on the economy to foster export. In the context of import, it is worth noting that monetary policy possesses the capacity to render import more economically viable through the manipulation of exchange rates. Fiscal policy has the potential to effectively mitigate the cost of imports and promote the facilitation of trade.

As a pivotal country within the BCIM-EC, Bangladesh plays a crucial role by leveraging its comparative advantages in bilateral import and export demand activities, as well as the utilization of its human and natural resources. Bangladesh should focus on exporting more goods to its three member trading countries by diversifying export goods, maintaining more capital-intensive goods than labor-intensive goods, seeking new openings, etc., and overall, an excellent friendly business policy should be implemented. However, the BCIM-EC will create a win-win situation among its member countries, especially Bangladesh, by ensuring regional and bilateral trade facilitation from its member countries.

Several limitations have the potential to influence the scope and accuracy of the findings. The presence of unobserved or exogenous factors could lead bias into the outcomes. Moreover, the inclusion of four adjacent neighboring countries may initiate regional heterogeneity. These limitations emphasize the importance of future research efforts when deriving further vision of inclusiveness for making policy decisions purely based on the findings of the study. In conclusion, to ensure sustainable bilateral regional trade among the BCIM-EC member countries, Bangladesh should prioritize the implementation of a well-balanced monetary and fiscal policy. These findings provide valuable insights for policymakers and stakeholders aiming to enhance trade relationships and economic cooperation within the BCIM-EC region.

Note.

1. The BCIM-EC, which encompasses Bangladesh, China, India, and Myanmar, Economic Corridor, as part of China's “Belt and Road Initiative”. Among the four countries involved, China is classified as an upper-middle-income country, whereas the remaining three countries are classified as lower-middle-income countries. The [46] discussed how close regional connectivity and the integration of the economy could benefit both countries, particularly in developing infrastructure and public policies to facilitate this process. The report emphasized the importance of developing infrastructure and executing efficacious public policies to accelerate this process and maximize the advantages it offers. Although the BCIM-EC is presently non-operational, its potential and scope are progressively expanding, in keeping with the wider phenomenon of globalisation. Facilitating regional connectivity plays a vital role in promoting economic growth and fostering cooperation among nations [48].
2 [49]. examined the implications of self-exciting threshold autoregressive (SETAR) models, while [50] explored the dynamics of nonlinear smooth transition autoregressive (STAR) models. We consider stationary nonlinear exponential STAR (ESTAR) in the study.

## Funding

This research was funded by the Study on the Construction of the Economic Belt in the Hexi Corridor, China (Grant number: 2022ZD009) and the study on the impact of green technology innovation on improving the quality of economic development--based on the perspective of green total factor productivity ( Grant number: 21lzujbkyxs009).

## 6 Data availability statement

Data will be made available on request.

## CRediT authorship contribution statement

**Arifur Rahman:** Writing – original draft, Writing – original draft, Methodology, Data curation, Conceptualization. **S. M. Woahid Murad** Writing – original draft, Software, Methodology, Investigation, Formal analysis. **Xiaowen Wang:** Writing – original draft, Validation, Supervision, Funding acquisition.

## Declaration of competing interest

The authors declare that they have no known competing financial interests or personal relationships that could have appeared to influence the work reported in this paper.
